# Comparison of registered and survey-based modes of HIV transmission in 2021–2023: Cross-sectional study in the Kyrgyz Republic

**DOI:** 10.1371/journal.pone.0330210

**Published:** 2025-08-19

**Authors:** Kostyantyn Dumchev, Anastassiya Stepanovich-Falke, Nikolay Lunchenkov, Anna Rohde, Anastasiya Danshyna, Aibek Bekbolotov, Aigul Solpueva, Aidana Kenzhekarieva, Aida Karagulova, Elmira Narmatova, Olga Varetska, Stela Bivol, Giorgi Kuchukhidze, Barbara Gunsenheimer-Bartmeyer

**Affiliations:** 1 Ukrainian Institute on Public Health Policy, Kyiv, Ukraine; 2 Robert Koch Institute, Berlin, Germany; 3 Technical University of Munich, TUM School of Social Sciences and Technology, Munich, Germany; 4 Republican Center for Viral Hepatitis und HIV/AIDS Control at the Ministry of Health of the Kyrgyz Republic, Bishkek, Kyrgyzstan; 5 Bishkek City AIDS Center, Bishkek, Kyrgyzstan; 6 Osh City AIDS Center, Osh, Kyrgyzstan; 7 The Global Fund, Geneva, Switzerland; 8 WHO Regional Office for Europe, Copenhagen, Denmark; Hormozgan University of Medical Sciences, IRAN, ISLAMIC REPUBLIC OF

## Abstract

**Background:**

Accurate identification of the mode of transmission (MoT) of HIV is critical for effective prevention. However, stigma associated with behaviors such as injecting drug use (IDU) and sex between men (MSM) can lead to misclassification of MoT data. This study replicates the methodology used in Ukraine to assess MoT misclassification and trends in Kyrgyzstan, with the aim of informing evidence-based epidemic control strategies.

**Methods:**

A cross-sectional survey was conducted among patients diagnosed with HIV in the six largest administrative units of Kyrgyzstan during the first three quarters of 2021–2023. The survey assessed pre-seroconversion HIV risk factors using self-administered, interviewer-assisted questionnaires, and HCV testing. The McNemar test compared registered and survey-based MoT, while logistic regression analyzed MoT trends over time.

**Results:**

A total of 1,962 new HIV diagnoses were registered in the study period, of them 480 individuals completed the survey. The proportion of cases attributable to IDU and MSM was higher in the survey than in the registration system (8.1% vs. 4.2%, p = 0.001 for IDU; 14.2% vs. 11.7% p = 0.12, for MSM), whereas heterosexual MoT was lower (76.0% vs. 80.2%, p = 0.038). Selling sex was reported by 2.9%, and in combination with IDU and MSM, 23.5% of participants could be categorized into one of the three key populations. An additional 18.1% belonged to bridge populations. There was a 23% increase in the absolute number of registered patients in the corresponding periods over three years, but the MoT distribution did not change.

**Conclusion:**

We found significant misclassification in IDU and heterosexual MoT, but not in MSM, possibly due to suboptimal survey sensitivity amid the increased stigmatization of the LGBTQI+ community. At least 41% of newly registered cases in Kyrgyzstan occurred in key and bridge populations, highlighting the need for intensified prevention efforts in these groups.

Understanding the mode of transmission (MoT) of HIV among newly diagnosed people living with HIV (PLHIV) is critical for monitoring the epidemic and targeting prevention efforts [1]. Assessment of MoT often relies on self-reporting of HIV risk behaviors by patients, which can be inaccurate, especially when associated with stigma or criminalization [2–4]. This is particularly true for injecting drug use (IDU), which is criminalized and stigmatized in many countries [5,6]. Stigma and discrimination against men who have sex with men (MSM) exist in many parts of the world [7,8], leading to underreporting of this behavior even in settings with increasing acceptance [9]. Misclassification of MoT in HIV surveillance systems may be exacerbated by the lack of standardized tools to collect sensitive information or by clinicians’ reluctance to inquire about behaviors that have no impact on patient management.

## Background

Surveys tend to be more effective in collecting data on sexual and substance use behavior [10] and it has been suggested that patients who report heterosexual acquisition of HIV and STIs should be regularly interviewed to determine their true risk factors [11]. Following this recommendation, a study conducted in Ukraine [12] found that the proportion of IDU and MSM modes of transmission was underestimated by 45% and 27%, respectively, while heterosexual MoT was overestimated by 74% among HIV patients registered in 2013–2015.

Kyrgyzstan (Kyrgyz Republic) is located in Central Asia, a subregion, where the HIV epidemic continues to grow [13]. In 2022, an estimated 10,000 adults were living with HIV, with a prevalence of 0.3% [14]. HIV and AIDS cases in Kyrgyzstan are monitored through a nationwide screening and routine case reporting system. From 2017 to 2021, a total of 3,829 HIV cases were reported, with no significant fluctuations observed over time [15]. During this period, 65% of cases were attributed to heterosexual transmission, 13% to injection drug use (IDU), and 7% to male-to-male sex. Notably, there was an increase in the proportion of cases attributed to heterosexual transmission (from 58% to 69%), a decrease in IDU-related cases (from 25% to 4%), and an increase in cases attributed to homosexual transmission (from 6% to 10%) over the same period.

However, case reporting data among MSM in the Kyrgyz Republic contrasts sharply with seroprevalence data collected through bio-behavioral surveys in 2021. A study in Bishkek that recruited 246 MSM found an HIV prevalence of 10.7%, slightly above 10.1% in 2016 [16]. Although reliable prevalence estimates were not available for other regions, extrapolation of the prevalence in Bishkek suggests that the true number of MSM living with HIV is likely much higher than the total number of patients registered with MSM as the mode of transmission, which was only 351. These data suggest significant underreporting and/or misclassification of MSM in the official case reporting system, likely due to their classification as heterosexual men. This may be due to social negative attitudes towards same-sex relationships and fear of stigmatization by healthcare providers. This assumption is supported by REAct, an international system for detection of human rights violations, which showed that nearly 38% cases on the combined basis of violation comprising sexual orientation and gender identity (SOGI) and HIV were registered in Kyrgyzstan in 2022, affecting primarily MSM, gay and bisexual men [17]. Additionally, data from a bio-behavioral survey among people who inject drugs in Kyrgyzstan also indicated that there is a substantial underreporting of IDU, particularly among women [18].

The current MoT registration system in the Kyrgyz Republic lacks clear guidelines and algorithms for risk categorization and reporting. Provider practices rely on individual understanding, resulting in non-standardized questioning and reporting of modes of transmission. There are also no clear recommendations for categorizing cases with multiple risks. Therefore, we hypothesized that there is a significant misclassification of MoT among the groups at higher risks of HIV infection in Kyrgyzstan in the official HIV surveillance system.

In this study, we replicated the methodology of the original Ukrainian study [12] to assess the extent of misclassification of key HIV modes of transmission (HET, MSM, and IDU) in the routine case registration system in the Kyrgyz Republic and to analyze trends in MoT distribution over time. The conclusions of the study are intended to inform the revision of the national guidelines for HIV risk assessment and MoT reporting and to improve the understanding of HIV epidemic trends.

## Methods

### Study design and sampling

We conducted a cross-sectional survey of people diagnosed with HIV from January to September in 2021 and 2022, and from January to August in 2023. To ensure national representation, we selected six administrative units with the largest populations: Bishkek city, Chui oblast, Jalal-Abad oblast, Osh oblast, Osh city, and Issyk-Kul oblast. The remaining three regions (Naryn, Batken, and Talass oblasts) were excluded due to the small number of registered cases, which would not significantly affect the study results.

The target sample size was 480 (160 per year), calculated a priori with 80% power and a 5% probability of type I error to detect 2% misclassification using a one-tailed McNemar test. The sample distribution among the six regions was proportional to the total number of registered cases (S1 Table).

Case registration data from the national HIV electronic medical information system [19] were used to sample patients. First, lists of patients registered each year were extracted from the system by authorized personnel (with only a numeric patient ID and no other personal identifiers) and filtered based on the following eligibility criteria: 18 years or older at diagnosis, a citizen of the Kyrgyz Republic, and not lost to follow-up. For sampling, the remaining list of eligible patients was randomly sorted using ‘RAND’ function in Microsoft Excel, and the required number of patient IDs were provided to clinical staff at each study site for recruitment. Recruitment procedures included contacting patients by telephone or in person during clinical visits, reading a standard study invitation script, and scheduling clinic appointments. Up to three contact attempts were made for each patient, with reasons for refusal or failure to reach the patient recorded.

### Data collection

All patients provided informed consent in a written form before completing a survey designed to collect information on potential HIV exposure. We used the structured questionnaire from the Ukrainian study with minimal adaptations. The questionnaire covered demographic information, HIV history, substance use patterns, sexual activity prior to seroconversion, and patients’ personal beliefs about how they acquired HIV.

The questionnaire was developed in Russian on the electronic platform e-DEN [16], which is commonly used for bio-behavioral surveys in Kyrgyzstan. The platform ensured data quality assurance, monitoring, and protection, with no personal identifiers included in the survey form to maintain confidentiality. The survey form excluded any personal identifiers to ensure confidentiality.

To minimize self-report bias on sensitive questions, we used a self-administered survey format. Respondents completed the survey on the e-DEN platform using a tablet. An interviewer was present in the same room to provide assistance and clarify questions, if needed. Interviewers, who represented non-governmental organizations with experience working with PLHIV, received special training in rapport building techniques. Throughout the consent process, it was emphasized that all responses would remain confidential and would not be shared with the clinic. On average, the entire survey process took 15–20 minutes.

After completion of the survey, patients without a record of HCV antibody testing or with a negative result older than 6 months were tested for HCV antibodies using an anti-HCV test (express RightSign HCV rapid test or ECOLAB ‘IFA-VHC’ anti-HCV enzyme immunoassay), depending on the availability of the test type at the study site.

## Data analysis

First, we performed descriptive analysis to examine the distribution patterns of reported modes of transmission (RMoT) in the medical information system and to assess the prevalence of various HIV risk factors. The electronic system classified RMoT into several categories: heterosexual (HET), men who have sex with men (MSM), injection drug use (IDU), blood product transfusion, organ or tissue transplant, other medical procedure, occupational exposure, other non-medical manipulation, and unknown. We excluded cases of mother-to-child transmission and grouped cases that fell outside of the primary categories of HET, MSM, and IDU into the ‘other’ category due to their negligible numbers.

In our study, we delineated each risk factor by creating binary variables from one or more relevant questions to indicate its presence or absence. These binary variables were formulated using logical expressions (S2 Table) to mitigate inconsistencies in participants’ responses regarding the same risk factor. In both our descriptive and hypothesis testing analyses, we treated these variables as non-mutually exclusive, recognizing that individuals could be exposed to multiple risk factors simultaneously. An additional variable was introduced to assess the ‘bridge’ population (defined as people who self-report heterosexual contacts with commercial sex workers, people who inject drugs [PWID], or PLHIV, and do not meet the criteria for key populations).

HCV infection, which is closely associated with IDU [20], was used as a proxy for the IDU risk factor among participants aged 45 years and older. This decision was underscored by the disproportionately high rates of anti-HCV positivity within this subgroup (22.4% in men and 10.3% in women, S3 Table), which contrasted sharply with the general prevalence in the Kyrgyz Republic, estimated at 2.6% [21], and taking into account extant evidence on HCV prevalence among PWID. In addition, given the increased susceptibility of MSM to certain sexually transmitted infections (STIs) [22], male respondents, who reported a history of oral or rectal gonorrhea, rectal herpes, or proctitis were classified as MSM.

We then created a summary variable to represent the most likely mode of transmission (SMoT) based on survey responses. In cases where only a single risk factor was identified, the corresponding SMoT category was assigned. For individuals with multiple risk exposures, the SMoT was assigned to the risk factor with the higher probability of transmission per act [23] and greater prevalence among key populations in Kyrgyzstan, following a predetermined hierarchy of mutually exclusive SMoT categories: IDU, MSM, HET, and Other (OTH).

There were no missing data in the data sets obtained from the electronic medical information system and e-DEN. Participants could refuse to answer sensitive questions, which was coded as ‘no response’. If either of the input variables were ‘no response’, the summary variable inherited the same value.

To assess the representativeness of our survey sample and the randomness of the sampling method, we used chi-squared tests to compare the distribution of RMoT between survey respondents and the remaining patients in the registry who were not included in the survey.

The primary objective of the analysis was to examine differences in the proportions of patients in corresponding RMoT and SMoT categories. Given the assessment of these variables in the same subjects, we used the McNemar test for paired proportions. To assess the significance of the trend in each SMoT proportion over time, we used univariable logistic regression with year (2021–2023) as a single numerical independent variable.

All analyses were performed using R version 4.3.2 [24].

### Ethics statements

All participants signed an informed consent form. Data collected through the e-DEN platform did not contain any personal identifiers. The study protocol, consent forms, and data collection instruments were approved by the Ethics Committee of the Ministry of Health of the Kyrgyz Republic by the ethical approval on 24 July 2023. All research methods were carried out according to relevant guidelines.

## Results

### Recruitment

A total of 1,962 new HIV diagnoses were registered in six study regions from January to September in 2021 and 2022, and from January to August in 2023 (S4 Table). We observed a 23% increase in the number of cases during the first six months of each year: from 419 in 2021–469 in 2022 and then to 515 in 2023.

Analysis of data from the electronic system showed that 4.2% of registered patients had died, 1.3% were either lost to follow-up or had moved out of the country, 6.8% were not citizens of the Kyrgyz Republic, and 2.9% were younger than 18 years of age. This left 1,665 patients, or 84.9% of those enrolled, who were eligible for our study. We contacted 857 patients to reach our target sample size. Of these, 503 patients, or 58.7%, agreed to participate. Recruitment was stopped when we successfully achieved our target sample size of 480 participants in all six regions. Recruitment rates – the percentage of patients recruited from those contacted – ranged from 44.2% to 97.3% across the different sites.

### Sample characteristics and risk factors

The recruited sample included 56.7% men and 43.3% women, with 66.7% identifying as Kyrgyz (Table 1). The median age was 38 years (IQR 30–47). The distribution of registered MoT in the sample was 80.2%, 4.2%, 11.7%, and 4% for HET, MSM, IDU, and unknown, respectively, which did not differ significantly from 377 patients who were eligible but were not recruited (S5 Table).

**Table 1:** Sociodemographic characteristics of the study participants.

		Total		Bishkek		Osh		Jalal-Abad oblast		Issyk-Kul Oblast		Osh oblast		Chuy oblast	
		N	%	N	%	N	%	N	%	N	%	N	%	N	%
Total		480		210		33		69		36		48		84	
Sex	male	272	56.7	116	55.2	18	54.5	40	58.0	24	66.7	32	66.7	42	50.0
	female	208	43.3	94	44.8	15	45.5	29	42.0	12	33.3	16	33.3	42	50.0
Ethnicity	Kyrgyz	320	66.7	163	77.6	14	42.4	37	53.6	31	86.1	20	41.7	55	65.5
	Uzbek	75	15.6	4	1.9	15	45.5	30	43.5			25	52.1	1	1.2
	Russian	62	12.9	32	15.2	1	3.0	1	1.4	3	8.3	1	2.1	24	28.6
	Dunganin	1	0.2							1	2.8				
	Tajik	1	0.2			1	3.0								
	Uyigur	11	2.3	5	2.4	2	6.1			1	2.8	2	4.2	1	1.2
	Other	10	2.1	6	2.9			1	1.4					3	3.6
Age category	18-24	39	8.1	15	7.1	2	6.1	5	7.2	2	5.6	12	25.0	3	3.6
	25-34	148	30.8	69	32.9	9	27.3	24	34.8	15	41.7	14	29.2	17	20.2
	35-44	148	30.8	69	32.9	12	36.4	21	30.4	5	13.9	9	18.8	32	38.1
	45+	145	30.2	57	27.1	10	30.3	19	27.5	14	38.9	13	27.1	32	38.1

The frequencies of each risk factor, as well as self-reported, registered, and survey-based MoT are presented with disaggregation by gender in **Table 2**. The data show that heterosexual sex is the predominant risk factor, reported by all surveyed women and 89.3% of men. Interestingly, nearly 20% of men reported engaging in male-to-male sexual activity. Injection drug use was self-reported by 23 men (8.5%) and only 2 women (1%). The prevalence of HCV antibodies was higher in men (12.5%) compared with women (4.8%), with marked differences by age and self-reported IDU status (S3 Table). Among participants who denied any history of IDU, HCV prevalence was 7.2% in men and 4.3% in women. In contrast, HCV prevalence was 69.6% among men who admitted to IDU use. Self-reported history of STIs was approximately 40% in both men and women, whereas selected oral/rectal STIs were more prevalent among men (11.4% compared to 4.8% in women). Commercial sex (selling sex for money or drugs) was reported by 2.9% of participants, with no gender difference. Based on these risk factors, 36.0% of men and 7.2% of women could be categorized into one of the three key populations (PWID, MSM or commercial sex workers). An additional 20.2% of men and 15.4% of women reported having sex with a PLHIV, PWID, or a sex worker, and thus can be classified as a bridge population. It is important to note the substantial level of non-response to either of the three questions defining this variable (113/480, 23.5%). Most of the non-responders (105/113, 92.9%) reported heterosexual exposure, suggesting an underestimation of the bridge population.

**Table 2:** HIV risk factors and modes of transmission by gender.

		Total		Men		Women	
		N	%	N	%	N	%
Injecting drug use (self-report)*	yes	25	5.2	23	8.5	2	1.0
	no	382	79.6	194	71.3	188	90.4
	no response	73	15.2	55	20.2	18	8.7
Injecting drug use (self-report & HCV)	yes	39	8.1	30	11.0	9	4.3
	no	368	76.7	187	68.8	181	87.0
	no response	73	15.2	55	20.2	18	8.7
Male-to-male sex (self-report)*	yes	54	11.3	54	19.9		
	no	426	88.8	218	80.1	n/a	
Male-to-male sex (self-report & rectal/oral STI)	yes	74	15.4	74	27.2		
	no	406	84.6	198	72.8	n/a	
Selling sex for money or drugs	yes	14	2.9	8	2.9	6	2.9
	no	452	94.2	250	91.9	202	97.1
	no response	14	2.9	14	5.1		
Heterosexual sex*	yes	452	94.2	244	89.7	208	100.0
	no	28	5.8	28	10.3		
Nosocomial risk*	yes	419	87.3	233	85.7	186	89.4
	no	21	4.4			21	10.1
	no response	40	8.3	39	14.3	1	0.5
Accidental risk*	yes	208	43.3	97	35.7	111	53.4
	no	258	53.8	165	60.7	93	44.7
	no response	14	2.9	10	3.7	4	1.9
STI history*	yes	192	40.0	108	39.7	84	40.4
	no	251	52.3	142	52.2	109	52.4
	no response	37	7.7	22	8.1	15	7.2
Rectal/oral STI history*	yes	41	8.5	31	11.4	10	4.8
	no	417	86.9	227	83.5	190	91.3
	no response	22	4.6	14	5.1	8	3.8
Key or bridge population*	key	113	23.5	98	36.0	15	7.2
	bridge	87	18.1	55	20.2	32	15.4
	neither	167	34.8	64	23.5	103	49.5
	no response	113	23.5	55	20.2	58	27.9
HCV status	negative	436	90.8	238	87.5	198	95.2
	positive	44	9.2	34	12.5	10	4.8
Registered MOT (RMoT)	IDU	20	4.2	17	6.3	3	1.4
	MSM	56	11.7	56	20.6	n/a	
	HET	385	80.2	188	69.1	197	94.7
	UNK	19	4.0	11	4.0	8	3.8
Self-reported MOT	ACC	19	4.0	6	2.2	13	6.3
	HET	240	50.0	115	42.3	125	60.1
	IDU	14	2.9	14	5.1		
	MSM	35	7.3	35	12.9	n/a	
	NOS	43	9.0	25	9.2	18	8.7
	UNK	129	26.9	77	28.3	52	25.0
Survey-based MOT (SMoT)	IDU	39	8.1	30	11.0	9	4.3
	MSM	68	14.2	68	25.0	n/a	
	HET	365	76.0	166	61.0	199	95.7
	OTH	8	1.7	8	2.9		

* Definitions of risk factors and the list of sexually transmitted infections is provided in S2 Table.

MOT, mode of HIV transmission; IDU, injecting drug use; MSM, male-to-male sex; HET, heterosexual; UNK, unknown; ACC, accidental; NOS, nosocomial; OTH, other.

### Degree of misclassification

Inclusion of anti-HCV positivity as an indicator of IDU among participants aged 45 years and older, and classification of male participants with selected STIs as MSM, changes our estimation of likely modes of transmission. With these adjustments, heterosexual transmission emerges as the most common probable MoT for men, accounting for 61%, followed by MSM at 25%, IDU at 11%, and other at 2.9%. For women, the analysis attributed 4.3% of cases to IDU, with the vast majority, 95.7%, attributed to heterosexual transmission.

**Table 3** compares RMoT and SMoT overall and by sex and age groups. Among men over 45, IDU was more commonly reported in the survey (25.4%) than in the registry (11.9%; p = 0.016), a discrepancy also seen in the overall sample. For women over 45, a similar difference was observed (10.3% vs. 2.6%), though not statistically significant. For men, HET was more frequent in the registry (69.1%) than in the survey (61.0%; p = 0.01), with a significant difference overall (p = 0.038).

**Table 3:** Comparison of the distribution of registered and survey-based modes transmission.

			Registered MOT		Survey-based MOT		McNemar statistic	McNemar p-value
			N	%	N	%		
women	18-24	IDU						
		HET	6	75.0	8	100.0	0.50	0.480
		UNK	2	25.0				
	25-34	IDU	1	2.0				
		HET	48	96.0	50	100.0	0.50	0.480
		UNK	1	2.0				
	35-44	IDU			1	1.4		
		HET	70	97.2	71	98.6	0.00	1.000
		UNK	2	2.8				
	45+	IDU	2	2.6	8	10.3	3.13	0.077
		HET	73	93.6	70	89.7	0.36	0.546
		UNK	3	3.8				
men	18-24	IDU			1	3.2		
		MSM	18	58.1	17	54.8	0.00	1.000
		HET	12	38.7	7	22.6	2.29	0.131
		OTH			6	19.4		
		UNK	1	3.2				
	25-34	IDU	1	1.0	4	4.1	1.33	0.248
		MSM	27	27.6	31	31.6	0.38	0.540
		HET	67	68.4	62	63.3	0.64	0.424
		OTH			1	1.0		
		UNK	3	3.1				
	35-44	IDU	8	10.5	8	10.5	0.00	1.000
		MSM	7	9.2	13	17.1	1.79	0.181
		HET	57	75.0	55	72.4	0.07	0.789
		UNK	4	5.3				
	45+	IDU	8	11.9	17	25.4	5.82	**0.016**
		MSM	4	6.0	7	10.4	0.57	0.450
		HET	52	77.6	42	62.7	4.05	**0.044**
		OTH			1	1.5		
		UNK	3	4.5				
Gender	women	IDU	3	1.4	9	4.3	2.50	0.114
		HET	197	94.7	199	95.7	0.06	0.814
		UNK	8	3.8				
	men	IDU	17	6.3	30	11.0	7.58	**0.006**
		MSM	56	20.6	68	25.0	2.42	0.120
		HET	188	69.1	166	61.0	6.68	**0.010**
		OTH			8	2.9		
		UNK	11	4.0				
Total		IDU	20	4.2	39	8.1	11.17	**0.001**
		MSM	56	11.7	68	14.2	2.42	0.120
		HET	385	80.2	365	76.0	4.30	**0.038**
		OTH			8	1.7		
		UNK	19	4.0				

MOT, mode of HIV transmission; IDU, injecting drug use; MSM, male-to-male sex; HET, heterosexual; UNK, unknown; OTH, other.

The crude estimate of MoT misclassification was substantial: the number of IDU-related cases was underestimated in the registration system by 48.7% (39 cases in the survey vs. 20), the number of MSM-related cases was underestimated by 17.6% (68 cases vs. 56), and heterosexual MoT was overestimated by 5.4% (365 cases vs. 385). To assess the sensitivity of the survey in detecting stigmatizing behaviors, we examined the concordance between RMoT and self-reported behaviors. This analysis revealed a substantial number of patients who were registered with MSM or IDU MoT but did not disclose respective behaviors in the survey (18 of 56 [32.1%] for MSM, 4 of 17 [23.5%] for IDU, S6 Table).

### Trends over time

**Fig 1:**
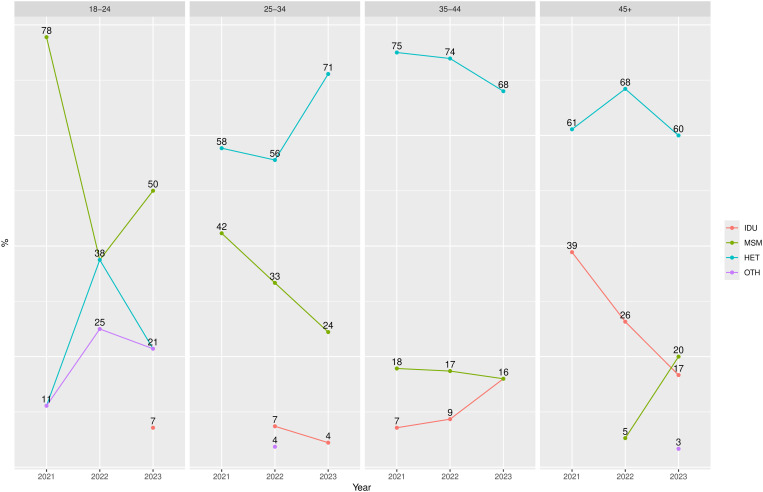
Trends in HIV modes of transmission among men in the study sample, by age and year of registration. Data points represent the percentage of respective survey-based mode of HIV transmission among men in the sample. IDU, injecting drug use; MSM, male to male sex; HET, heterosexual; OTH, other.

In the analysis of SMoT trends over time, the proportion of HET increased from 61.7% in 2021 to 71.1% in 2023 among men, and from 93.3% to 96.2% among women, but these changes were not significant (Table 4). The proportion of IDU also did not change, but the absolute number of newly registered cases was low, particularly among women. In the age subgroup analysis (Fig 1), only one marginally significant increase was observed—the proportion of MSM among men older than 45 rose from 5.3% to 20% (p = 0.063)

**Table 4:** Trends in survey-based modes of transmission in the study sample.

		MOT	Year of registration						Trend coef.	Trend p-value
			2021		2022		2023			
			N	%	N	%	N	%		
women	18-24	HET	2	100.0	2	100.0	4	100.0		
	25-34	HET	14	100.0	19	100.0	17	100.0		
	35-44	IDU	1	5.0					−18.10	0.997
		HET	19	95.0	23	100.0	29	100.0	18.10	0.997
	45+	IDU	3	12.5	2	8.0	3	10.3	−0.11	0.816
		HET	21	87.5	23	92.0	26	89.7	0.11	0.816
men	18-24	IDU					1	7.1	17.57	0.997
		MSM	7	77.8	3	37.5	7	50.0	−0.51	0.248
		HET	1	11.1	3	37.5	3	21.4	0.23	0.659
		OTH	1	11.1	2	25.0	3	21.4		
	25-34	IDU			2	7.4	2	4.4	0.52	0.461
		MSM	11	42.3	9	33.3	11	24.4	−0.41	0.118
		HET	15	57.7	15	55.6	32	71.1	0.32	0.210
		OTH			1	3.7				
	35-44	IDU	2	7.1	2	8.7	4	16.0	0.47	0.307
		MSM	5	17.9	4	17.4	4	16.0	−0.07	0.859
		HET	21	75.0	17	73.9	17	68.0	−0.17	0.574
		OTH								
	45+	IDU	7	38.9	5	26.3	5	16.7	−0.58	0.093
		MSM			1	5.3	6	20.0	1.82	0.063
		HET	11	61.1	13	68.4	18	60.0	−0.05	0.873
		OTH					1	3.3		
Gender	men	IDU	9	11.1	9	11.7	12	10.5	−0.03	0.883
		MSM	23	28.4	17	22.1	28	24.6	−0.09	0.587
		HET	48	59.3	48	62.3	70	61.4	0.04	0.783
		OTH	1	1.2	3	3.9	4	3.5		
	women	IDU	4	6.7	2	2.9	3	3.8	−0.32	0.448
		HET	56	93.3	67	97.1	76	96.2	0.32	0.448
Total		IDU	13	9.2	11	7.5	15	7.8	−0.09	0.653
		MSM	23	16.3	17	11.6	28	14.5	−0.06	0.708
		HET	104	73.8	115	78.8	146	75.6	0.04	0.751
		OTH	1	0.7	3	2.1	4	2.1		

MOT, mode of HIV transmission; IDU, injecting drug use; MSM, male-to-male sex; HET, heterosexual; UNK, unknown; OTH, other.

## Discussion

In our study, we assessed the accuracy of HIV transmission categories within a nationally representative sample of recently diagnosed PLHIV in Kyrgyzstan using a sensitive survey methodology. The methodology was adapted from a similar study conducted in Ukraine and included a standardized assessment of HIV risk factors and HCV seroprevalence as a biological marker of injecting drug use, as well as selected STIs as a possible biological indicator of homosexual exposure.

Our analysis revealed substantial underreporting of IDU as a mode of transmission in the official registration system, where IDU was documented in 4.2% of cases compared to the 8.3% identified in our sample. This notable discrepancy was largely due to the detection of HCV antibodies, suggesting a greater frequency of IDU transmission than that identifiable by self-report. The remarkable rate of non-response to questions related to IDU in our questionnaire also supports this finding: 73 participants, or 15% of the total, did not provide definitive answers to one or more questions in this section. This pattern suggests a tendency among newly diagnosed PLHIV to withhold information about IDU, both in our survey and during their initial clinic registration, likely influenced by the prevailing stigma and fear of criminalization associated with drug use in Kyrgyzstan [8,25]. The reluctance to disclose IDU may be particularly pronounced among women, as suggested by a recent study examining the dynamics of sexual partners among PWID in Kazakhstan and Kyrgyzstan [18]. Furthermore, the extent of IDU misclassification in our study was consistent with findings from a previous study conducted in Ukraine. It’s worth noting, however, that the proportion of IDU-related cases identified in Ukraine was higher (33.2% in registration data versus 59.7% in survey responses), highlighting the varying magnitude of this problem in different contexts [12].

Our second major finding is the overestimation of heterosexual transmission of HIV among men, as evidenced by the registration data indicating a prevalence of 69.1% compared to the 61.0% found in our survey. This discrepancy resulted in a downward revision of the estimated prevalence of HET transmission in the total sample from 80.2% to 76%.

We faced a notable challenge in accurately assessing the misclassification of the MSM mode of transmission, largely due to the extensive underreporting of homosexual experience. This survey coincided with the period of time leading up to the enactment of a national law prohibiting the dissemination of information about LGBTQI+ rights, which was signed into law by the President of Kyrgyzstan in August 2023. The prevailing political discourse, which has been contributing to the stigmatization of the LGBTQI+ community, likely influenced the performance of the survey as a method for collecting sensitive information [26].

Our findings revealed that approximately 30% of men who reported male-to-male sex were not classified as MSM in official records. Conversely, 32% of those recorded as MSM did not report homosexual experience in our survey. These results suggest that, while our survey method was more effective than the registration system in detecting cases of male-to-male sex for some, it was less effective for others. To address potential underestimation of MSM-related cases, particularly those disproportionately affected by certain STIs [27], we classified individuals who reported a history of certain infections (such as rectal and oral gonorrhea, proctitis, and rectal herpes) as MSM [22]. However, due to the potential for heterosexual transmission, syphilis was excluded from this criterion, despite a higher prevalence of syphilis reported among men than women in our study. This exclusion likely contributed to the underestimation of the proportion of MSM in our sample, given the higher prevalence of syphilis among MSM worldwide [28]. As a result of these complexities, we were unable to provide a precise quantitative estimate of MSM misclassification. In comparison, the Ukrainian study reported that 17% of men identified with the MSM mode of transmission did not disclose homosexual experience, suggesting that the level of non-disclosure in Kyrgyzstan may be higher, possibly due to increased social stigma and the fear of prosecution and broader individual consequences [29].

In our sample, a combination of IDU, MSM and commercial sex could account for 23.5% of HIV transmission. This represents a conservative minimal estimate of the proportion of key populations among new HIV diagnoses in Kyrgyzstan. Additionally, approximately 18% could be attributed to sexual contacts with key populations, placing them in the category of a bridge population. This figure is notably lower than the estimates produced by UNAIDS models for Eastern Europe and Central Asia in 2022. These models suggest that the proportion of new cases occurring among clients of sex workers and sex partners of key populations ranges from 23% [13] to 50% [30]. However, a direct comparison is not entirely valid, due to our focus on newly registered rather than true incident cases. Nonetheless, the observed difference may be partially explained by the likely underestimation in our study (indicated by a high non-response rate to these questions), the epidemiological differences between Kyrgyzstan and the rest of the region, and the growing proportion of bridge populations among new cases, confirmed by the recent study [30]. The latter factor poses a theoretical risk of an HIV epidemic expansion to the general population in Kyrgyzstan, emphasizing the need for further surveillance and in-depth research.

We observed a marked increase in the number of newly registered HIV cases over the three study years – with 23% more diagnoses in the first six months of 2023 compared to 2021. However, this trend may not reflect the situation in all regions of Kyrgyzstan [31], and may be partially explained by the post-COVID compensatory increase in HIV testing as well as HIV testing through donor-funded projects in certain areas. Trends in the distribution of modes of transmission were not statistically significant, suggesting that the overall increase in new HIV diagnoses is not due to one particular MoT. On the other hand, in the context of increasing stigma and more hostile legislation [32,33], it is possible MSM may become more reluctant to disclose homosexuality, and the true proportion of MSM-related cases may remain stable or even increase.

It is important to note that the absolute numbers of newly registered IDU and MSM cases did not decrease over the examined period 2021–2023. This indicates that transmission within the PWID and MSM populations continues at a sustainable level, posing a risk of HIV outbreaks, if prevention programs in these key populations are scaled down, as was seen in other countries [34,35]. The existing sentinel surveillance system should be vigilant to detect such outbreaks and intervene early. Importantly, stigma towards PWID and MSM in Kyrgyzstan remains a significant facilitator of HIV transmission and a barrier to prevention and treatment [25]. Stigma reduction interventions should be implemented at multiple levels to improve access to prevention services and prevent outbreaks.

Our study has several limitations. As discussed above, the most important one is the suboptimal sensitivity of the survey in detecting behaviors such as IDU, male-to-male sex, and commercial sex in the context of the restrictive legal environment, pervasive stigma and criminalization, and an increasingly conservative tenor of the political discourse. We took all feasible measures to develop rapport with the participants and increase the accuracy of self-report, such as using private rooms for the study procedures, self-administration of questionnaire, assistance by independent interviewers trained in counseling techniques, assurances of full confidentiality of survey responses, and de-personalized questionnaire form. To further mitigate this factor, we used anti-HCV prevalence as a marker of IDU-related transmission. Due to the low HCV seropositivity among patients younger than 45, we were unable to compare the prevalence to that of the general population. Therefore, we decided to limit this extrapolation to the older subgroup, which may contribute to underestimation of IDU in our analysis among the younger age groups. With the same purpose, we applied oral/rectal STI history as a marker of homosexual exposure. Nevertheless, we acknowledge that the degree of underreporting might have been non-negligible and was probably higher than in the Ukrainian study, likely due to the differences in the cultural and political context, which should be studied in more detail.

Another limitation arises from the inability of survey-based methods to establish a mode of transmission when multiple risk factors are present. We used a hierarchy based on the probability of transmission per act and population prevalence to assign the most probable modes of transmission, but this does not exclude a possibility for example for heterosexual women to be infected through nosocomial exposure or drug-injecting MSM to be infected via homosexual contact.

## Conclusion

In the study of HIV modes of transmission in the Kyrgyz Republic we found a significant misclassification of injecting drug use and heterosexual modes of transmission. The magnitude of misclassification was lower than in the earlier Ukrainian study, due to the lower number of IDU and MSM cases relative to the heterosexual category, a different cultural context and a hostile political climate, evidenced by higher non-disclosure of stigmatized behaviors. The accuracy of the HIV case registration system may be improved by introducing standardized tools for risk factor ascertainment, taking into account HCV seroprevalence and STI history as markers of risk exposures.

Another important finding was the increasing number of newly registered cases, primarily attributed to heterosexual exposure, calling for additional prevention efforts, particularly among bridge populations. Other key categories, namely IDU and MSM, remained stable in absolute numbers, and therefore should not be de-prioritized in prevention programming.

Continued enhancement of the surveillance system coupled with a comprehensive understanding of transmission dynamics are vital for advancing HIV prevention and achieving sustained progress in controlling the spread of the HIV in the Kyrgyz Republic in line with the WHO Global Health Sector Strategies on, respectively, HIV, viral hepatitis and sexually transmitted infections for the period 2022–2030 [36].

## Supporting information

S1 Table Selection of regions and target sample size. (DOCX)

S2 Table Logical formulas for defining risk behaviors. (DOCX)

S3 Table HCV seroprevalence by sex, age, and self-reported injecting drug use. (DOCX)

S4 Table Number of registered HIV cases and recruitment process. (DOCX)

S5 Table Comparison of the registered mode of transmission between the recruited sample and not recruited eligible patients. (DOCX)

S6 Table Cross-tabulation of reported mode of transmission and risk behaviors reported in the survey among men. (DOCX)

S1 File HIV Modes of Transmission study Participant Questionnaire. (DOCX)
