# Supplementary material for: Comparison of registered and survey-based modes of HIV transmission in 2021–2023: Cross-sectional study in the Kyrgyz Republic
Source: PLoS One. 2025 Aug 19;20(8):e0330210. doi: 10.1371/journal.pone.0330210 (PMC12364321; doi:10.1371/journal.pone.0330210)
Supplement: S1 Table — (DOCX) [file pone.0330210.s001.docx]

Supplementary Table S1. Selection of regions and target sample size

|  | **Region** | **Number of HIV cases diagnosed in 2021** | **Proportion of HIV cases diagnosed in 2021** | **Target sample size** |
| --- | --- | --- | --- | --- |
|  | Bishkek city | 347 | 41% | 210 |
|  | Chui obl. | 145 | 17% | 84 |
|  | Jalal-abad obl. | 112 | 13% | 69 |
|  | Osh obl. | 78 | 9% | 48 |
|  | Issyk-kul obl. | 58 | 7% | 36 |
|  | Osh city | 52 | 6% | 33 |
|  | Naryn obl. | 19 | 2% |  |
|  | Batken obl. | 18 | 2% |  |
|  | Talass obl | 14 | 2% |  |
|  | **Total** | **843** | **100%** | **480** |
