# Supplementary material for: Comparison of registered and survey-based modes of HIV transmission in 2021–2023: Cross-sectional study in the Kyrgyz Republic
Source: PLoS One. 2025 Aug 19;20(8):e0330210. doi: 10.1371/journal.pone.0330210 (PMC12364321; doi:10.1371/journal.pone.0330210)
Supplement: S2 Table — (DOCX) [file pone.0330210.s002.docx]

Supplementary Table S2. Logical formulas for defining risk behaviors

| Heterosexual | heterosexual contacts (regardless of having homosexual contacts) OR having one or more partners of the opposite sex OR self-reporting being infected through heterosexual contact |
| --- | --- |
| Injecting drug use | injecting any illicit drug at least once OR self-reporting injecting drug use as the most likely way of acquiring HIV |
| Homosexual | being a male AND (having sexual contacts with men at the present time OR having had one or more male sexual partners OR having sexual partner of the same sex OR self-reporting MSM as the most likely way of acquiring HIV) |
| Nosocomial | having had blood or blood product transfusion OR having had organ or tissue transplantation OR having had *in vitro* fertilization OR self-reporting being infected through medical procedures |
| Accidental | intentional skin penetration (tattoo, scarring, other practices) OR having been exposed to blood of another person through damaged skin or mucosa OR self-reporting being infected in an occupational or non-occupational accident with skin penetration |
| STI | history of hepatitis B OR gonorrhea OR syphilis OR genital herpes OR rectal herpes OR venereal warts OR other STI |
| Rectal or oral STI | history of rectal gonorrhea OR oral gonorrhea OR rectal herpes OR proctitis |
| Bridge population | (NOT reporting injecting drug use [defined above] AND NOT reporting homosexual exposure [defined above] AND NOT being positive for anti-HCV antibodies AND NOT reporting history of rectal or oral STIs AND NOT reporting selling sex for money or drugs) AND (having had a sexual partner who injects drugs OR having had a sexual contact with a person living with HIV OR having had a sexual partner who was bisexual) |

All factors are assessed by self-report, for the period of 10 years before the first positive HIV test.

STI, sexually transmitted infection.
