# Supplementary material for: Comparison of registered and survey-based modes of HIV transmission in 2021–2023: Cross-sectional study in the Kyrgyz Republic
Source: PLoS One. 2025 Aug 19;20(8):e0330210. doi: 10.1371/journal.pone.0330210 (PMC12364321; doi:10.1371/journal.pone.0330210)
Supplement: S3 Table — (DOCX) [file pone.0330210.s003.docx]

Supplementary Table S3. HCV seroprevalence by sex, age, and self-reported injecting drug use.

|  |  |  | **Total** | | **Injecting drug use** | | | |  |  |
| --- | --- | --- | --- | --- | --- | --- | --- | --- | --- | --- |
|  |  | **HCV status** | **N** | **%** | **yes** | | **no** | | **no response** |  |
|  |  |  |  |  | **N** | **%** | **N** | **%** | **N** | **%** |
| female | 18-24 | neg | 8 | 100.0 |  |  | 8 | 100.0 |  |  |
|  | 25-34 | neg | 49 | 98.0 |  |  | 45 | 100.0 | 4 | 80.0 |
|  |  | pos | 1 | 2.0 |  |  |  |  | 1 | 20.0 |
|  | 35-44 | neg | 71 | 98.6 | 1 | 100.0 | 64 | 98.5 | 6 | 100.0 |
|  |  | pos | 1 | 1.4 |  |  | 1 | 1.5 |  |  |
|  | 45+ | neg | 70 | 89.7 |  |  | 63 | 90.0 | 7 | 100.0 |
|  |  | pos | 8 | 10.3 | 1 | 100.0 | 7 | 10.0 |  |  |
| male | 18-24 | neg | 27 | 87.1 | 1 | 100.0 | 21 | 91.3 | 5 | 71.4 |
|  |  | pos | 4 | 12.9 |  |  | 2 | 8.7 | 2 | 28.6 |
|  | 25-34 | neg | 94 | 95.9 | 3 | 75.0 | 63 | 96.9 | 28 | 96.6 |
|  |  | pos | 4 | 4.1 | 1 | 25.0 | 2 | 3.1 | 1 | 3.4 |
|  | 35-44 | neg | 65 | 85.5 | 1 | 12.5 | 56 | 94.9 | 8 | 88.9 |
|  |  | pos | 11 | 14.5 | 7 | 87.5 | 3 | 5.1 | 1 | 11.1 |
|  | 45+ | neg | 52 | 77.6 | 2 | 20.0 | 40 | 85.1 | 10 | 100.0 |
|  |  | pos | 15 | 22.4 | 8 | 80.0 | 7 | 14.9 |  |  |
| Sex | female | neg | 198 | 95.2 | 1 | 50.0 | 180 | 95.7 | 17 | 94.4 |
|  |  | pos | 10 | 4.8 | 1 | 50.0 | 8 | 4.3 | 1 | 5.6 |
|  | male | neg | 238 | 87.5 | 7 | 30.4 | 180 | 92.8 | 51 | 92.7 |
|  |  | pos | 34 | 12.5 | 16 | 69.6 | 14 | 7.2 | 4 | 7.3 |
| Total |  | neg | 436 | 90.8 | 8 | 32.0 | 360 | 94.2 | 68 | 93.2 |
|  |  | pos | 44 | 9.2 | 17 | 68.0 | 22 | 5.8 | 5 | 6.8 |
