# Supplementary material for: Comparison of registered and survey-based modes of HIV transmission in 2021–2023: Cross-sectional study in the Kyrgyz Republic
Source: PLoS One. 2025 Aug 19;20(8):e0330210. doi: 10.1371/journal.pone.0330210 (PMC12364321; doi:10.1371/journal.pone.0330210)
Supplement: S4 Table — (DOCX) [file pone.0330210.s004.docx]

Supplementary Table S4. Number of registered HIV cases and recruitment process

|  |  | **N** | **%** |
| --- | --- | --- | --- |
| Year and quarter of registration | 2021 Q1 | 202 | 10.3 |
|  | 2021 Q2 | 217 | 11.1 |
|  | 2021 Q3 | 170 | 8.7 |
|  | 2022 Q1 | 211 | 10.8 |
|  | 2022 Q2 | 258 | 13.1 |
|  | 2022 Q3 | 240 | 12.2 |
|  | 2023 Q1 | 272 | 13.9 |
|  | 2023 Q2 | 243 | 12.4 |
|  | 2023 July-August | 149 | 7.6 |
|  | **Total** | **1962** |  |
| Eligibility | deceased | 83 | 4.2 |
|  | deregistered | 25 | 1.3 |
|  | not KR citizen | 133 | 6.8 |
|  | age<18 | 56 | 2.9 |
|  | eligible | 1665 | 84.9 |
|  | **Total** | **1962** |  |
| Contact attempt | no | 808 | 48.5 |
|  | yes | 857 | 51.5 |
|  | **Total** | **1665** |  |
| Number of contact attempts | 1 | 600 | 70.0 |
|  | 2 | 155 | 18.1 |
|  | 3 | 102 | 11.9 |
|  | **Total** | **857** |  |
| Final result of contact attempts | moved outside of KR | 61 | 7.1 |
|  | moved inside KR | 65 | 7.6 |
|  | lost to follow-up | 14 | 1.6 |
|  | refused care | 18 | 2.1 |
|  | deceased | 42 | 4.9 |
|  | contact info invalid | 81 | 9.5 |
|  | unable to attend | 19 | 2.2 |
|  | able to attend | 503 | 58.7 |
|  | refused to participate | 47 | 5.5 |
|  | other | 7 | 0.8 |
|  | **Total** | **857** |  |
| Successfully recruited | no | 377 | 44.0 |
|  | yes | 480 | 56.0 |
|  | **Total** | **857** |  |

KR, Kyrgyz Republic.
