# Supplementary material for: Comparison of registered and survey-based modes of HIV transmission in 2021–2023: Cross-sectional study in the Kyrgyz Republic
Source: PLoS One. 2025 Aug 19;20(8):e0330210. doi: 10.1371/journal.pone.0330210 (PMC12364321; doi:10.1371/journal.pone.0330210)
Supplement: S5 Table — (DOCX) [file pone.0330210.s005.docx]

Supplementary Table S5. Comparison of the registered mode of transmission between the recruited sample and not recruited eligible patients.

| **Registered MoT** | **Successfully recruited** | | | | **Chi-square** | **p-value** |
| --- | --- | --- | --- | --- | --- | --- |
|  | **no** | | **yes** | |  |  |
|  | **N** | **%** | **N** | **%** |  |  |
| HET | 299 | 79.3 | 385 | 80.2 | 0.06 | 0.811 |
| IDU | 7 | 1.9 | 20 | 4.2 | 2.97 | 0.085 |
| MSM | 48 | 12.7 | 56 | 11.7 | 0.14 | 0.712 |
| UNK | 23 | 6.1 | 19 | 4.0 | 1.65 | 0.200 |
| Total | 377 |  | 480 |  |  |  |

MoT, mode of HIV transmission; IDU, injecting drug use; MSM, male-to-male sex; HET, heterosexual; UNK, unknown; ACC, accidental; NOS, nosocomial.
