# Supplementary material for: Comparison of registered and survey-based modes of HIV transmission in 2021–2023: Cross-sectional study in the Kyrgyz Republic
Source: PLoS One. 2025 Aug 19;20(8):e0330210. doi: 10.1371/journal.pone.0330210 (PMC12364321; doi:10.1371/journal.pone.0330210)
Supplement: S6 Table — (DOCX) [file pone.0330210.s006.docx]

Supplementary Table S6. Concordance between registered mode of transmission and risk behaviors reported in the survey among men.

|  |  | **Registered MoT** | | | | | |
| --- | --- | --- | --- | --- | --- | --- | --- |
|  |  | IDU | Col% | not IDU | Col% | Total | Col% |
|  |  | Row % |  | Row% |  | Row% |  |
| **Self-reported injecting drug use** | yes | 13 | 76.5% | 10 | 3.9% | 23 | 8.5% |
|  |  | 56.5% |  | 43.5% |  | 100.0% |  |
|  | no | 4 | 23.5% | 190 | 74.5% | 194 | 71.3% |
|  |  | 2.1% |  | 97.9% |  | 100.0% |  |
|  | no response | 0 | 0.0% | 55 | 21.6% | 55 | 20.2% |
|  |  | 0.0% |  | 100.0% |  | 100.0% |  |
|  | total | 17 | 100.0% | 255 | 100.0% | 272 | 100.0% |
|  |  | 6.3% |  | 93.8% |  | 100.0% |  |
|  |  |  |  |  |  |  |  |
|  |  | MSM | Col% | not MSM | Col% | Total | Col% |
|  |  | Row% |  | Row% |  | Row% |  |
| **Self-reported male-to-male sex** | yes | 38 | 67.9% | 16 | 7.4% | 54 | 19.9% |
|  |  | 70.4% |  | 29.6% |  | 100.0% |  |
|  | no | 18 | 32.1% | 200 | 92.6% | 218 | 80.1% |
|  |  | 8.3% |  | 91.7% |  | 100.0% |  |
|  | total | 56 | 100.0% | 216 | 100.0% | 272 | 100.0% |
|  |  | 20.6% |  | 79.4% |  | 100.0% |  |

MoT, mode of HIV transmission; IDU, injecting drug use; MSM, male-to-male sex.
