# Supplementary material for: Comparison of registered and survey-based modes of HIV transmission in 2021–2023: Cross-sectional study in the Kyrgyz Republic
Source: PLoS One. 2025 Aug 19;20(8):e0330210. doi: 10.1371/journal.pone.0330210 (PMC12364321; doi:10.1371/journal.pone.0330210)
Supplement: S1 File — (DOCX) [file pone.0330210.s007.docx]

Supplementary Material. HIV Modes of Transmission study Participant Questionnaire

| Date | \|  \|  \|  \|  \|  \|  \|  \|  \|  \|  \| \| --- \| --- \| --- \| --- \| --- \| --- \| --- \| --- \| --- \| --- \| |
| --- | --- | --- | --- | --- | --- | --- | --- | --- | --- | --- | --- |
| Respondent ID | \|  \|  \|  \|  \|  \|  \|  \|  \|  \|  \|  \| \| --- \| --- \| --- \| --- \| --- \| --- \| --- \| --- \| --- \| --- \| --- \| |
| Gender *(circle the necessary)* | \| m \|  \| f \| \| --- \| --- \| --- \| |

**COMMENT 1:** In this survey, we are going to ask you questions about things you may have experienced such as medical procedures, drug use, travel and sexual activities. When you answer questions, please give me the best and most complete answer you can.

Next questions will be about your medical history. On these and other questions, I will be particularly interested in experiences you have had in the last 10 years, but only up until the time you were diagnosed with HIV infection (Mo/Yr of diagnosis ___/ ___/___ ___).

***Note to interviewer****: If respondent does not immediately recall, prompt with questions such as: Where were you living? Who were you living with? Were you working [or in school]? Continue probing until respondent recalls events as of 10 years ago. Once these are recalled, comment, "Keep in mind that I will be interested in experiences BEGINNING at that time, but only up until you were diagnosed with HIV infection."*

1. In the last 10 years and before your diagnosis of HIV infection did you receive any of the following procedures:

|  | Yes | No | Unknown | Refused to answer |
| --- | --- | --- | --- | --- |
| 1.1 A blood transfusion or blood products such as clotting factors |  |  |  |  |
| 1.2 Did you receive organ transplant (such as a kidney, heart or lung transplant, cornea transplant or bone graft)? |  |  |  |  |
| 1.3 Have you been hospitalized for any reason ? |  |  |  |  |
| 1.4. Did you have any type of surgery? |  |  |  |  |
| 1.5. Have you been on dialysis? |  |  |  |  |
| 1.6. Did you receive acupuncture? |  |  |  |  |
| 1.7. Did you receive dental services? |  |  |  |  |
| 1.8FEMALES: did you receive artificial insemination to try become pregnant? |  |  |  |  |

2. In the last 10 years and before your diagnosis of HIV infection did you receive an injection with a needle from a non-health care professional?

Yes No Unknown Refused to answer

3. In the last 10 years and before your diagnosis of HIV infection did you engage in any other activities involving blood, needles, or knives (for example, tattooing, scarring, or religious activities, manicure procedures)?

Yes No Unknown Refused to answer

4. In the last 10 years and before your diagnosis of HIV infection did you have any medical condition, which required frequent injections (for example, diabetes)?

Yes No Unknown Refused to answer

5. In the last 10 years and before your diagnosis of HIV infection did you receive or self-inject any substance with an unsterile or previously-used needle?

Yes No Unknown Refused to answer

**COMMENT 2**: In this survey, I am going to ask you questions about possible illnesses that you may have had in the past ten years. When you answer questions, please give me the best and most complete answer you can.

6. Did you have any of the following illnesses in the last 10 years but before your diagnosis with HIV infection?

|  | Yes | No | Unknown | Refused to answer |
| --- | --- | --- | --- | --- |
| 6.1. Hepatitis A (Infectious hepatitis) |  |  |  |  |
| 6.2. Hepatitis B (Serum hepatitis) |  |  |  |  |
| 6.3. Hepatitis C (Non-A, Non- B) |  |  |  |  |
| 6.4. Hepatitis, not sure what kind |  |  |  |  |
| 6.5. Gonorrhea (oral or pharyngeal) |  |  |  |  |
| 6.6. Rectal gonorrhea |  |  |  |  |
| 6.7. Nonspecific Urethritis (NGU) |  |  |  |  |
| 6.8. Syphilis |  |  |  |  |
| 6.9. Genital Herpes Simplex |  |  |  |  |
| 6.10 Rectal Herpes |  |  |  |  |
| 6.11 Venereal Warts |  |  |  |  |
| 6.12 Proctitis |  |  |  |  |
| 6.13 Any other sexually transmitted disease (such as genital ulcers, pelvic inflammatory disease, chancroid, lymphogranuloma venereum, trichomonas, chlamydia, etc.) |  |  |  |  |

**COMMENT 3**: The next questions relate to "street drugs," or drugs which were not prescribed by a doctor, as well as places where these drugs are found.

7. Did you visit a drug "shooting gallery" at any time in the last 10 years until the time you were diagnosed with HIV infection?

Yes No Unknown Refused to answer

8. Did you use by injection or non-injection any of the following drugs at any time in the last 10 years until the time you were diagnosed with HIV infection?

|  |  | Injection | | Non-injection | | Unknown | Refused to answer |
| --- | --- | --- | --- | --- | --- | --- | --- |
|  |  | Yes | No | Yes | No |  |  |
| **Opiates** | | | | | | | |
| 8.1 | Heroin |  |  |  |  |  |  |
| 8.2 | Hanka |  |  |  |  |  |  |
| 8.3 | Methadone (crystal) |  |  |  |  |  |  |
| 8.4 | Other, *specify* |  |  |  |  |  |  |
| **Stimulants** | | | | | | | |
| 8.5 | Cocaine |  |  |  |  |  |  |
| 8.6 | Mephedrone |  |  |  |  |  |  |
| 8.7 | Amphetamine (‘fen’) (in the form of powder) |  |  |  |  |  |  |
| 8.8 | Methamphetamine powder (crystal) |  |  |  |  |  |  |
| 8.9 | ‘Bath salts’ |  |  |  |  |  |  |
| 8.10 | Pharmacy drugs (tropicamide, rinazolin, calypsol, ketamine)  ***Drugs, which are purchased at drugstores and which do not need special preparation for use for therapeutic purposes*** |  |  |  |  |  |  |
| 8.11 | Methyl​enedioxy​methamphetamine (MDMA, ecstasy) |  |  |  |  |  |  |
| 8.12 | Tramadol/tramal |  |  |  |  |  |  |
| 8.13 | Other, *specify* |  |  |  |  |  |  |

**COMMENT 4**: The next questions are related to your sexual activities. All questions refer to the time period in the last 10 years but before you were diagnosed with HIV infection. Sexual contact refers to vaginal, anal, or oral sex with either a man or a woman.

9. How many female partners did you have sexual contact with in the last 10 years but before you were diagnosed with HIV infection? ________

10. How many male partners did you have sexual contact with in the last 10 years but before you were diagnosed with HIV infection? ________

***Note to interviewer****: If respondent answered NONE (0) to Questions 10* ***and*** *11, GO TO* ***Question 17***

11. In the last 10 years but before you were diagnosed with HIV infection which of the following types of sexual contact did you participate in? (Check all that apply)

Vaginal _____ Anal _____ Oral _____

12. In the last 10 years but before you were diagnosed with HIV infection did you have sex with person who injected drugs?

Yes No Unknown Refused to answer

13. In the last 10 years but before you were diagnosed with HIV infection did you have sex with person who is known to have AIDS or HIV infection?

Yes No Unknown Refused to answer

14. During this time period (*in the last 10 years but before you were diagnosed with HIV infection)*, did you have sexual contact with a sex worker (someone who exchanges sex for drugs or money)?

Yes No Unknown Refused to answer

15. During the time period in the last 10 years but before you were diagnosed with HIV infection, did you ever get paid, or receive drugs, for sex?

Yes No Unknown Refused to answer

16. Did you ever get vaccinated against hepatitis B?

Yes No Unknown Refused to answer

17. How do you think you contracted the HIV virus?

___________________________________________________________________________

____________________________________________________________________________

**COMMENT 5**: Thank you for your time in completing this survey. The information will be very helpful to us in learning more about HIV infection.
